# Cognitive function in older patients and their stress challenge using different anesthesia regimes: a single center observational study

**DOI:** 10.1186/s12871-022-01960-7

**Published:** 2023-01-06

**Authors:** Soeren Wagner, Martin Breitkopf, Elena Ahrens, Haobo Ma, Olivia Kuester, Christine Thomas, Christine A. F. von Arnim, Andreas Walther

**Affiliations:** 1 Department of Anesthesiology and Intensive Care, School of Medicine, Technical University of Munich, Klinikum rechts der Isar, Ismaninger Straße 22, 81675 Munich, Germany; 2 Department of Anesthesia, Critical Care and Pain Medicine, Beth Israel Deaconess Medical Center, Harvard Medical School, Boston, MA USA; 3 Department of Anesthesiology and Intensive Care, Katharinenhospital Klinikum Stuttgart, Stuttgart, Germany; 4 Department of Neurology, Universitaetsklinikum Ulm, Ulm, Germany; 5 Department of Old Age Psychiatry and Psychotherapy, Klinikum Stuttgart, Krankenhaus Bad Cannstatt, Stuttgart, Germany; 6 Department of Geriatrics, University of Goettingen Medical School, Goettingen, Germany

**Keywords:** Cognitive function, Stress, Cortisol

## Abstract

**Background:**

With increasing age older patients are at higher risk for cognitive decline after surgery. Even tailored anesthesia procedures in older patients remain a high risk for postoperative cognitive disorder. Additional stress derived from anxiety and anesthesia itself can negatively impact postoperative cognitive outcomes. The objective of this study was to evaluate the impact of general versus regional anesthesia on postoperative cognitive disorder and indicators of perioperative stress in elderly undergoing surgery.

**Methods:**

In this single center prospective study between December 2014 and November 2015, 46 patients aged 50 to 85 years undergoing dermatology surgery were enrolled. Patients were stratified by receiving general versus regional nerve anesthesia. On three consecutive days, saliva cortisol levels were analyzed three times per day. Cognitive function was assessed on the day before and the day after surgery using comprehensive neuropsychological testing of multiple cognitive functions including memory, executive function, attention and processing speed.

**Results:**

Comparing the regional anesthesia group (RAG, *n* = 28) with the general anesthesia group (GAG, *n* = 18) no significant difference in the postoperative cognitive function was observed. However, patients in the GAG had significantly higher postoperative cortisol levels when compared to patients in the RAG. In both groups, a peak of cortisol value was detected on the day of surgery, which was higher in the GAG in comparison to the RAG.

**Conclusions:**

We did not observe a difference in postoperative cognitive function between patients undergoing regional or general anesthesia for dermatology surgery. However, we found lower cortisol level in the RAG. Based on these findings, future studies should investigate alternatives to reduce stress in a general anesthesia setting.

**Trial registration:**

ClinicalTrials.gov ID: NCT02505815.

## Background

With an ageing society, health care providers worldwide face similar difficulties encountering older patients. Besides multi-organ dysfunction, older patients are at higher risk for cognitive decline, challenging the medical and surgical treatment of this ageing population. Cognition requires numerous components of cortical and subcortical domains like memory, attention and executive function in order to identify, perceive and solve problems and to generate future knowledge and experience [1]. Therefore, the cognitive integrity of older patients is of pivotal importance in everyday life for the activities of daily living (ADL) and quality of life (QOL). Anesthesia procedures in older patients pose a high risk for postoperative cognitive dysfunction regardless of their current preserved cognitive function [2, 3]. In elderly patients, these symptoms are detected more frequently compared to younger patients after general anesthesia [4]. Moreover, the diminished cognitive flexibility persists longer and in its severe occurrence can lead to permanent postoperative cognitive disorder (POCD). Anxiety is a well-known phenomenon in elderly surgical patients and has a pivotal impact on perioperative stress. Additional stress derived from anesthesia influences postoperative physical and cognitive outcomes [5], leading to an increase in POCD and impairment in elderly surgical patients with an incidence of over 45% [6, 7]. Thus, accurately planned anesthesia procedures could help to reduce perceived stress and might therefore attenuate any negative impact on cognitive postoperative function. Risk factors for the development of POCD include age, low educational level and preexisting reduced cognitive reserve [2, 3]. Moreover, the anesthesia procedure can play an important role, but current understanding of what technique carries the lowest risk is unknown. Some studies suggested that procedures with regional are more recommended in comparison to general anesthesia to avoid POCD. However, this could not be proven yet [8]. The combination of elevated stress level and regional anesthesia are believed to increase POCD in elderly patients. This observation leads to the question if a general anesthesia is associated with lower stress level and consequently translates into a reduced risk of POCD. In the present study, we investigated the association between general versus regional anesthesia in patients undergoing dermatology surgery and postoperative cognitive disorder. We further investigated whether a general versus regional anesthesia was associated with post-procedural cortisol levels.

## Methods

In this single-center observational study, two patient arms were compared; the general anesthesia group (GAG) vs. the regional anesthesia group (RAG). This study was conducted at the University Hospital of Erlangen, Germany between December 2014 and November 2015 in accordance with the guidelines for Good Clinical Practice and the Declaration of Helsinki. The study approval was given by the local Ethics committee (Ethikkommission der Medizinischen Fakultät der Friedrich-Alexander-Universität Erlangen-Nürnberg, Erlangen, Germany) (reference number: 245_14 B, on 18.09.2014) (ClinicalTrials.gov ID: NCT02505815). Each patient read and signed a consent form before included in the study.

### Patients

After written informed consent, 48 patients were enrolled to the study. Inclusion criteria were age between 50 and 85 years, an American Society of Anesthesiologist (ASA) physical status less than IV as well as an estimated postoperative stay of at least two days after receiving either regional or general anesthesia. Patients with a history of neurological or neuropsychological deficits or diseases including Cushing’s disease, stroke or epilepsy, or with prescriptions of neuropsychological medication were excluded from the study. Moreover, patients with alcohol and illicit drug abuse, time shifts within the test protocol and patients undergoing cardiac surgery were not included into the study. Moreover, patients with perioperative medication like benzodiazepine treatment and any glucocorticoids were not included into the study. Patients susceptible to postoperative nausea and vomiting (PONV) were excluded from this study. In view of the fact that a maximum of three patients can be tested sensibly every day so that the test phases took place in the same period and do not take place in the evening hours, the respective groups were gradually completed in one after the other. However, as a result, more patients were asked than were ultimately included in the study. To avoid a systematic bias all patients have been enrolled in the department of dermatology for a comparable duration and invasiveness of surgery. Malignant neoplasms of the skin and subcutis were excised at different parts of the body. These include melanomas, basalcell carcinomas and diseased skin and subcutaneous tissue with as yet uncertain pathology, which were mainly found on the scalp and facial skin. The primary disease had no influence or effects on the cortisol parameters or the cognitive function of all the study patients. Moreover, the examiners were blinded to the scheduled form of anesthesia.

### Cortisol

In order to quantify a biomarker for the individual stress level, patients submitted a saliva sample at three specified times of the day on the day prior to surgery, then on the day of surgery and first postoperative day. Prior to salvia collection, patients were instructed to not brush their teeth, rinse their mouth, smoke or eat. The cortisol daily profile was determined from these saliva samples. For this purpose, in the morning immediately after waking up, at noon and in the evening, swabs (Salivette→ Sarstedt, Nümbrecht, Germany) were moistened with saliva by means of chewing. Patients were instructed to adhere to the same time of day with the saliva collection. On the day of surgery, the second probe was collected immediately before surgery and postoperatively after transfer from the postoperative care facility at a similar time as the other day before. Saliva samples were temporarily stored in the refrigerator at 2–4° Celsius and then transferred to be stored at -80° Celsius until analysis. Cortisol concentrations were determined by liquid chromatography tandem mass spectrometry as previously described at the University Hospital of Erlangen [9–11].

### Cognitive assessment

In order to assess potential alterations in patients’ cognitive function, a cognitive assessment was carried out at two different time points using a neuropsychological test assessment [12, 13]. The baseline testing was recorded on the day before surgery and the second survey on the first postoperative day. To ensure comparability, the schedule of both assessments was synchronized. Furthermore, numerous test systems are used in the literature, so that comparability is still difficult. We aimed to evaluate as many different cognitive domains as possible with our assessment. To avoid learning effects, only instruments with available parallel versions were applied. To avoid fatigue, test time was limited to a maximum half an hour. The neuropsychological test battery was composed as follows:

We used the DemTect test as psychomotor screening test to detect mild cognitive functional impairments [14]. The DemTect test assess “verbal memory”, “verbal fluency”, “cognitive flexibility”, and “attention” in five consecutive, separate tasks while accounting for age and education. To evaluate the global memory function we used the Rivermead Behavioral Memory test (RBMT) [15]. To assess the patient’s short term memory capacity we used the digit span forward test and its backwards variant the digit span backward test for verbal working memory (digit span backward test) [16]. The cognitive domains of attention, concentration and executive function were addressed with the Stroop test [17]. Finally, due to its nine-subtest structure, memory and attention deficits were by the SKT test [18]. Further details have been previously described [12, 13]. Table 1 summarizes cognitive assessments and corresponding tested cognitive function and domain.

**Table 1:** Cognitive assessment and corresponding tested cognitive function and domain

Assessment	Domain
DemTect [14]	Cognitive impairment screening test
RBMT [15]	Global memory function
Wechsler Memory Scale Digit span forward [16]	Attention, short-term memory
Wechsler Memory Scale Digit span backwards [16]	Attention, working memory
Stroop [17]	Processing speed, attention, executive function
SKT [18]	Memory and attention screening test

*SKT* Short cognitive performance test, *RBMT* Rivermead behavioral memory test

### Clinical Protocol

After participants were included in the study, their medication plans were documented and it was ensured that no chronic medication was prescribed that had a modulating effect on neurocognitive function. At the same time, questions were asked about previous experiences with PONV, since no corticoids may be administered in the study protocol. Thus, PONV susceptible patients were not included in the study. For premedication, benzodiazepines were strictly avoided in all patients and instead, if necessary, clonidine 75 or 150 µg was given preoperatively. Total intravenous anesthesia with Remifentanil and Propofol was chosen in the GAG and managed as target controlled infusion (TCI). Before placing a laryngeal mask, the patients received 0.1 mg Fentanyl for anesthesia induction and additionally weight-adapted Rocuronium was administrated for endotracheal intubation as a neuromuscular blocking agent. During the entire surgery, vital data were monitored and in particular attention was paid to perioperative hemodynamic stability in order to avoid hypotensive phases. We defined a more than 20% decrease from preoperative baseline awake systolic blood pressure measurement as a hypotensive phase. Moreover, hypotension defined as systolic blood pressure lower than 80 mmHg was avoided by administration of 1-2-ml Theodrenalin-Cafedrin. In addition, patients’ peripheral oxygen saturation was monitored. Patients were pre-oxygenated with 100% oxygen before induction of anesthesia until the end-tidal oxygen concentration reached values higher than 80%, and patients received 100% oxygen during emergence before extubation, likewise. After extubation, patients were transferred to recovery room. Oxygen was given during the transfer and was continued in the recovery room as to institutional standards. In the RAG, patients received local anesthetic for the surgery area with Lidocaine 1% s.c. Vital data were also monitored in this group and oxygen was insufflated by simple face mask or nasal cannula. These patients were also transferred to the recovery room for postoperative monitoring. Both groups received 7.5 mg to 15 mg piritramide over 20 min depending on total body weight for pain therapy, if pain severity of 4 points or more on an 11-point numerical pain scale was evaluated.

### Data management and statistical analysis

Statistical analysis was performed using IBM SPSS Statistics version 27.0 for macOS platforms. Outliers were identified using the Grubbs test and were not included in further data analysis. To test normality for scale variables, the Kolmogorov-Smirnov test and the Shapiro-Wilks test were used. The Mann-Whitney U test was used to compare the mean scores of both groups based on age and cortisol levels. Spearman’s test was used to analyze the correlation between age and cortisol levels. Fisher’s exact test was used to compare the differences in risk factors between the two groups and a univariate ANOVA was performed to analyze the influence of risk factors on cortisol levels.

Comparing the differences in cognitive tests between the two groups, the Mann-Whitney U test was used. Due to the various tests, the level of significance was set at *p* < 0.005 for the two-factor variance-analysis, whereas it was defined at *p* < 0.05 for all other statistical tests.

## Results

**Fig. 1:**
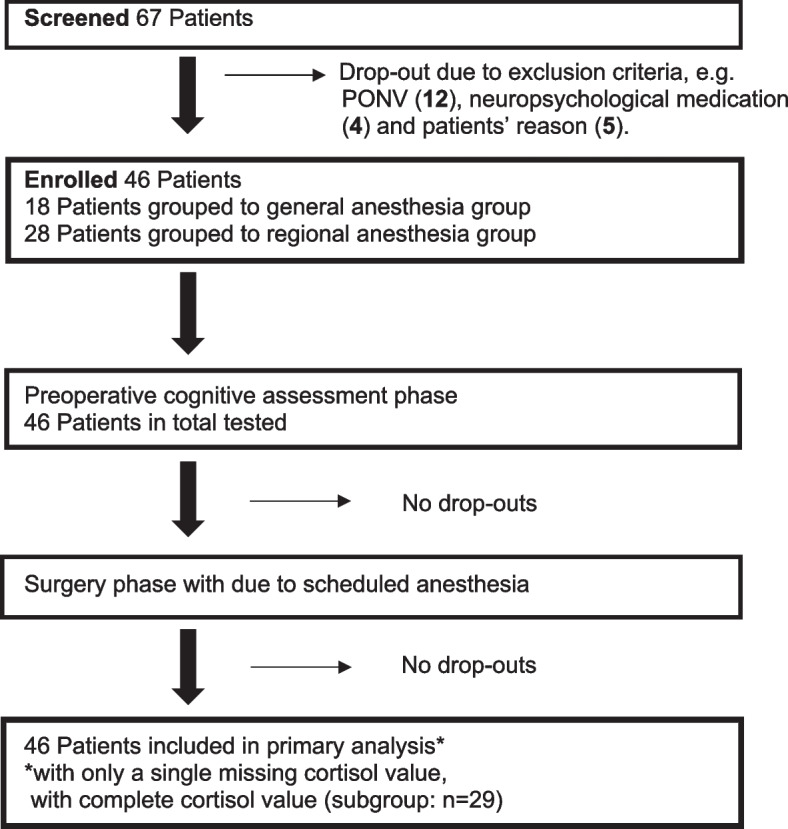
Patients’ recruitment and follow-up flow chart. *PONV* postoperative nausea and vomiting

We screened 67 patients for eligibility and had to exclude 12 patients from participating in the study because of PONV, 4 patients due to neuropsychological medication and 5 patients regarding individual personal reasons. Overall, 48 patients were included in the study, out of which 2 patients did not complete the test phase. Reasons for discontinuing the study varied in nature, such as discomfort after surgery or pain, or missing cortisone samples (Fig. 1) 

Demographic and clinical data are given in Table 2.

**Table 2:** Patients’ data

**Demographics** **(*n*** = 46**)**	**RAG** **(*n*** = 28**)**	**GAG (*n*** = 18**)**	***p*** **value**
Age (years)	75.4 (62–85)	62.0 (52–75)	< 0.001*
Sex (male/female)	17/11	15/3	0.095
Height (cm)	170 (156–186)	175 (154–190)	0.042*
Body weight (kg)	78 (71–103)	90.5 (54–120)	0.031*
BMI (kg/m^2^)	29.3 (22.3–37.8)	30.05 (22.8–28.4)	0.489
ASA physical status	2.46 (2–3)	1.83 (1–2)	< 0.001*
Anesthesia time (minutes)	39 (24–52)	93.5 (39–223)	< 0.001*
**Risk factors**	**n/28 (%)**	**n/18 (%)**	***p*** **value**
Arterial hypertension	21 (75)	7 (39)	0.029*
Diabetes mellitus	8 (29)	5 (17)	0.486
Hyperlipoproteinemia	14 (50)	9 (50)	0.99
Alcohol abuse	9 (32)	7 (39)	0.754
Nicotine abuse (x > 5 per day)	23 (82)	9 (50)	0.047*
**Chronic medication**	**n/28 (%)**	**n/18 (%)**	***p*** **value**
Beta blocker	13 (46)	5 (17)	0.058
ACE inhibitor	6 (21)	1 (6)	0.220
AT III antagonists	12 (43)	2 (11)	0.027*
Diuretics	12 (43)	1 (6)	0.007*
Insulin or metformin	7 (25)	2 (11)	0.448
Statins	9 (32)	5 (17)	0.315
Thyroid medication	2 (7)	2 (11)	0.639
Antiplatelet drugs	11 (39)	5 (17)	0.188
Calcium antagonists	8 (29)	2 (11)	0.274

Data are reported as absolute number, mean (range) or as percentage. Risk factors and chronic medication are listed. A *p* value < 0.05 was considered as statistically significant

*BMI* Body mass index, *ASA* American Society of Anesthesiologists, *ACE* Angiotensin-converting enzyme, *RAG* Regional anesthesia group, *GAG* General anesthesia group

The mean age in the RAG was 75.4 years. In the GAG the mean value was 62.0 years. In the RAG 17 out of 28 patients were male, whereas 15 out of 18 patients were male in the GAG, with no statistically significant difference (*p* = 0.095). Level of significance (*p = 0.05). However, the ASA value was significantly different between both groups, with a higher value in the RAG. Moreover, the duration of anesthesia was longer and nicotine abuse was higher in the RAG.

### Comparison cortisol level in GAG and RAG

Cortisol levels were determined on three consecutive days at three fixed timepoints. Individual cortisol values out of the complete set of cortisol measurement on a single day were missing for 17 patients. This single missing cortisol measurement for each of these patients occurred completely randomly and distributed throughout the study. Therefore, the entire cohort was analyzed in a first step, and the same analysis was carried excluding the patients with missing values in a second step. A comparison of the mean values in this subgroup analysis showed the same result with a statistically significant difference in each case. All cortisol values from both groups are presented in Table 3.

The mean value of the total cortisol level was 5.47 U/L in the GAG and 2.56 U/L in the RAG (*n* = 46, *p* = 0.006). The mean value in the subgroup (*n* = 29) of the total cortisol level was 6.36 U/L in the GAG and 2.35 U/L in the RAG (*p* = 0.028).

### Comparison cortisol level on individual days

The mean cortisol values were consistently higher on all three days in patients receiving general anesthesia compared to patients receiving regional anesthesia (Table 3). The analysis of the entire cohort revealed similar results as after excluding patients with at least one missing cortisol value one any of the three days. Moreover, a mixed ANOVA revealed similar pattern cortisol values on any of the three days over all patients.

The cortisol values and the analysis of the subgroup (*n* = 29) excluding individuals with a missing value on any of the three days can be found in Table 3. This analysis revealed a similar pattern as the analysis in all patients.

**Table 3:** Average cortisol data for three consecutive days in both groups

	1st day Cortisol (U/L)	2nd day Cortisol (U/L)	3rd day Cortisol (U/L)	Total Cortisol (U/L)
**Entire cohort**				
RAG (*n* = 28)	3.39 (SD 2.59)	1.48 (SD 1.96)	1.78 (SD 2.84)	2.56 (SD 2.59)
GAG (*n* = 18)	7.41 (SD 3.81)	4.61 (SD 5.09)	4.56 (SD 3.75)	5.47 (SD 3.46)
Difference, *p* value	0.045*	0.021*	0.027*	0.006*
**After excluding patients with missing data**				
RAG (*n* = 21)	2.20 (SD 2.2)	2.57 (SD 2.0)	2.29 (SD 2.39)	2.35 (SD 2.03)
GAG (*n* = 8)	5.87	7.49	5.72	6.36
Difference, *p* value	0.013*	0.041*	0.047*	0.028*

Data are presented as mean (standard deviation [SD]). Note that within this data analysis at least a single cortisol value out of the complete measurement set is missing (see limitations). The lower part of the table presents a full set of cortisol data analysis. A *p* value < 0.05 was considered as statistically significant

*RAG* Regional anesthesia group, *GAG* General anesthesia group

### Correlation of age and cortisol level

There was no correlation between age and total cortisol level in either group (GAG: *p* = 0.78 and RAG: *p* = 0.422).

### Distribution of risk factors for cortisol release perioperatively and selected chronic medication

We analyzed how often a risk factor occurred in the respective group and whether this difference was statistically significant. The risk factors frequencies are related to the respective group and the check for statistical significance are listed in Table 2. There were more patients with arterial hypertension and nicotine abuse in the RAG. We analyzed how often a certain chronic medication was taken by the patient. The distribution of the frequency and the respective difference between the two groups is shown in Table 2.

There was a statistically significant difference observed in the RAG group for higher proportion of patients taking AT III antagonists and more diuretics than in the GAG. Moreover, the age was significantly higher in the RAG in comparison to GAG.

### Influence of risk factors and chronic medication on cortisol level

In exploratory analyses, we investigated the association between individual risk factors of POCD and cortisol levels. There was no association between a history of arterial hypertension, nicotine abuse, the use of AT III antagonists or diuretics and POCD (Table 4).

**Table 4:** ANOVA for total cortisol level and corresponding risk factor in both groups

Risk factors / chronic medication	RAG *p* value	GAG *p* value
Arterial hypertension	0.222	0.458
Nicotine abuse	0.617	0.287
AT III antagonists	0.723	0.719
Diuretics	0.688	0.131

*RAG* Regional anesthesia group, *GAG* General anesthesia group

### Differences in cognitive tests pre- and postoperatively and comparison of both groups

Each test of the neurocognitive assessment was analyzed preoperatively and postoperatively. When comparing both groups, the FWIT test showed a better result in the RAG group for both assessment phases. The RMBT test showed a better result preoperatively in the GAG. The SKT memory test and the Digit Span backwards test showed a better result in the GAG postoperatively.

However, between the two groups the differences between the pre- and postoperative results in the neurocognitive testing showed no significant difference in any of the tests. By comparing the test result differences, itself, we elicit only in one test system a significant difference (see Table 5). However, the digital span test forward and digital span test backwards itself were not significantly different. Thus, only if both tests are analyzed together, there is a significant difference (see Table 6).

**Table 5:** Patients’ test results

Test	Preoperative			Postoperative			Difference		
	RAG (*n* = 28)	GAG (*n* = 18)	*p* value*	RAG (*n* = 28)	GAG (*n* = 18)	*p* value*	RAG (*n* = 28)	GAG (*n* = 18)	*p* value*
**FWIT (sec)**	34.02 (21–70)	26.30 (16–44)	0.009*	31.71 (18–57)	25.94 (15–40)	0.012*	-2.32 (-13–10)	-0.36 (-6–5)	0.242
**DemTect (points)**	13.04 (7–18)	13.72 (10–17)	0.533	12.18 (8–18)	13.28 (5–18)	0.174	-0.86 (-7–6)	-0.44 (-6–4)	0.497
**RBMT (points)**	21.21 (8–39)	26.67 (14–42)	0.017*	19.00 (9–32)	22.50 (11–30)	0.080	-2.21 (-14–9)	-4.17 (-14–3)	0.299
**SKT total (points)**	3.00 (0–9)	2.94 (0–9)	0.828	3.43 (0–10)	3.22 (0–9)	0.927	0.43 (-3–6)	0.28 (-3–3)	0.991
**SKT attention (points)**	2.21 (0–7)	1.56 (0–9)	0.072	2.64 (0–10)	1.44 (0–6)	0.112	0.43 (-4–4)	-0.11 (-3–2)	0.409
**SKT memory (points)**	0.79 (0–4)	1.39 (0–3)	0.057	0.79 (0–3)	1.78 (0–4)	0.005*	0 (-4–2)	0.39 (-2–3)	0.503
**Digit Span total** **(points)**	11.79 (5–17)	14.33 (8–22)	0.092	12.14 (6–18)	14.00 (9–22)	0.335	0.36 (-5–4)	-0.33 (-2–3)	0.159
**Digit Span forward (points)**	7.07 (3–12)	8.00 (3–12)	0.152	7.11 (3–11)	7.56 (3–12)	0.654	0.04 (-4–3)	-0.44 (-3–2)	0.110
**Digit Span backwards (points)**	4.71 (2–7)	6.33 (2 − 11)	0.075	5.04 (3–8)	6.44 (3–11)	0.047*	0.32 (-3–3)	0.11 (-2–2)	0.498

Data are reported as mean (range). Shown data represent the difference between both group assessment values by comparing the differences between pre- and postoperative values within a group. A *p* value < 0.05 was considered as statistically significant

*RAG* Regional anesthesia group, *GAG* General anesthesia group, *SKT* Short cognitive performance test, *FWIT* Color-word-interference-test, *RBMT* Rivermead behavioral memory test

**Table 6:** Difference in test results between the groups

Test	Difference		
	RAG (*n* = 28)	GAG (*n* = 18)	*p* value*
**FWIT (sec)**	-2.32 (-8–10)	-0.36 (-6–5)	0.091
**DemTect (points)**	-0.86 (-7–6)	-0.44 (-6–4)	0.549
**RBMT (points)**	-2.21 (-9–9)	-4.17 (-9–3)	0.142
**SKT total (points)**	0.43 (-3–6)	0.28 (-3–3)	0.666
**SKT attention (points)**	0.43 (-4–4)	-0.11 (-3–2)	0.962
**SKT memory (points)**	0.01 (-4–2)	0.39 (-2–3)	0.212
**Digit Span total** **(points)**	0.36 (-5–4)	-0.33 (-2–3)	0.014*
**Digit Span forward (points)**	0.04 (-4–3)	-0.44 (-3–2)	0.433
**Digit Span backwards (points)**	0.32 (-3–3)	0.11 (-2–2)	0.118

Data are reported as mean (range). Shown data represent the difference between both subgroup assessment values by comparing the differences between pre- and postoperative values within a group. A *p* value < 0.05 was considered as statistically significant

*RAG* Regional anesthesia group, *GAG* General anesthesia group, *SKT* Short cognitive performance test, *FWIT *Color-word-interference-test, *RBMT* Rivermead behavioral memory test

After finding a significant difference in patient age between the two groups, we performed an age matched subgroup analysis using a range of three years of age around the calculated mean value. Results are presented in Table 7 and demonstrate significant differences in anesthesia time and nicotine abuse. However, these statistical differences have been demonstrated in the entire cohort before. Moreover, we consecutively performed a subgroup analysis for the parameter of height, weight, ASA physical status and duration of anesthesia. There was no statistically significant result within these subgroups.

**Table 7:** Subgroup patients’ data

Demographics (*n* = 26)	RAG (*n* = 17)	GAG (*n* = 9)	*p* value
Age (years)	71.4 (62–79)	68.7 (59–75)	0.164
Sex (male/female)	11/6	8/1	0.201
Height (cm)	171 (160–186)	173 (154–184)	0.518
Body weight (kg)	85.2 (75–103)	85.1 (54–104)	0.606
BMI (kg/m^2^)	29.5 (22.3–37.8)	28.1 (22.8–28.4)	0.699
ASA physical status	2.2 (2–3)	2.3 (1–2)	0.699
Anesthesia time (minutes)	40.8 (33–52)	89.6 (39–136)	0.007*
**Risk factors**	**n/17 (%)**	**n/9 (%)**	***p*** **value**
Arterial hypertension	12 (71)	6 (67)	0.845
Diabetes mellitus	4 (24)	3 (33)	0.609
Hyperlipoproteinemia	9 (53)	5 (56)	0.905
Alcohol abuse	7 (71)	5 (56)	0.504
Nicotine abuse (x > 5 per day)	1 (6)	4 (44)	0.017*
**Chronic medication**	**n/17 (%)**	**n/9 (%)**	***p*** **value**
Beta blocker	5 (29)	2 (22)	0.708
ACE inhibitor	4 (24)	1 (11)	0.465
AT III antagonists	6 (35)	2 (22)	0.512
Diuretics	7 (71)	1 (11)	0.123
Insulin or metformin	3 (18)	2 (22)	0.789
Statins	5 (29)	2 (22)	0.708
Thyroid medication	0 (0)	1 (11)	0.178
Antiplatelet drugs	5 (29)	2 (22)	0.071
Calcium antagonists	6 (35)	2 (22)	0.512

Data are reported as absolute number, mean (range) or as percentage. Risk factors and chronic medication are listed. A *p* value < 0.05 was considered as statistically significant

*BMI* Body mass index, *ASA *American Society of Anesthesiologists, *ACE* Angiotensin-converting enzyme, *RAG* Regional anesthesia group, *GAG* General anesthesia group

Regarding the cortisol level the subgroup analysis elicit no significant differences between both groups. However, the cortisol level in the RAG were tendentially still lower than in the GAG (see Table 8).

**Table 8:** Subgroup patients’ average cortisol data for three consecutive days

Entire cohort	1st day Cortisol (U/L)	2nd day Cortisol (U/L)	3rd day Cortisol (U/L)	Total Cortisol (U/L)
RAG (*n* = 17)	4.07	1.58	1.65	2.66
GAG (*n* = 9)	6.103	3.845	4.02	4.86
Difference, p value	0.407	0.401	0.156	0.058

Data are presented as mean. A *p* value < 0.05 was considered as statistically significant

*RAG* Regional anesthesia group, *GAG* General anesthesia group

Moreover, we performed a subgroup analysis regarding the cognitive assessment results. However, only in a single test section we found a significant difference between both groups (see Table 9).

**Table 9:** Subgroup patients’ test results

Test	Preoperative			Postoperative			Difference		
	RAG (*n* = 17)	GAG (*n* = 9)	*p* value*	RAG (*n* = 17)	GAG (*n* = 9)	*p* value*	RAG (*n* = 17)	GAG (*n* = 9)	*p* value*
**FWIT (sec)**	39.60 (21–43)	27.44 (20–44)	0.367	27.99 (18–38)	27.17 (20–40)	0.426	-1.61 (-12–3)	-0.27 (-6–5)	0.491
**DemTect (points)**	13.94 (7–18)	14.11 (11–17)	0.672	13.06 (8–18)	14.00 (13–15)	0.597	-0.88 (-7–6)	-0.11 (-3–2)	0.339
**RBMT (points)**	22.65 (10–39)	24.44 (18–31)	0.241	21.47 (9–32)	21.00 (11–29)	0.916	-1.18 (-10–9)	-3.44 (-3–3)	0.792
**SKT total (points)**	2.59 (0–9)	2.56 (1–7)	0.833	3.12 (0–8)	3.00 (1–5)	0.792	0.53 (-3–6)	0.53 (-3–3)	0.634
**SKT attention (points)**	1.94 (0–6)	1.33 (0–6)	0.220	2.29 (0–6)	1.22 (0–3)	0.367	0.35 (-4–4)	-0.12 (-3–2)	0.525
**SKT memory (points)**	0.65 (0–4)	1.22 (0–3)	0.164	0.82 (0–3)	1.78 (0–4)	0.045*	0.18 (-4–2)	0.56 (-2–3)	0.916
**Digit Span total** **(points)**	12.41 (5–17)	12.78 (8–19)	0.999	12.00 (6–17)	12.67 (9–18)	0.672	-0.41 (-5–3)	-0.12 (-2–3)	0.999
**Digit Span forward (points)**	7.24 (3–12)	7.12 (3–10)	0.874	6.65 (3–11)	6.78 (3–10)	0.751	-0.59 (-4–2)	-0.33 (-2–1)	0.999
**Digit Span backwards (points)**	5.18 (2–7)	5.67 (3–9)	0.999	5.35 (3–8)	5.89 (4–8)	0.396	0.18 (-3–2)	0.22 (-2–2)	0.999

Data are reported as mean values (range). Shown data represent the difference between both subgroup assessment values by comparing the differences between pre- and postoperative values within a subgroup. A *p* value < 0.05 was considered as statistically significant

*RAG* Regional anesthesia group, *GAG* General anesthesia group, *SKT* Short Cognitive performance test, *FWIT* Color-word-Interference-test, *RBMT* Rivermead behavioral memory test

## Discussion

The aim of this study was to evaluate the association of different anesthesia strategies (regional versus general anesthesia) with postoperative stress, indicated by cortisol levels and cognitive outcome in elderly patients. When comparing the two study groups we did not find any differences in the development of early postoperative cognitive disorder. Moreover, there was no significant difference in the baseline testing values when comparing age matched pairs.

However, we observed that patients receiving general anesthesia had higher postoperative cortisol levels at each of the scheduled days. It is also noticeable that in both groups we found a peak of cortisol value on the day of surgery, which was more pronounced in the GAG in comparison to the RAG.

In addition, the average patient age was significantly higher in the regional anesthesia group. We assume that due to a higher patient age as well as an expected shorter duration of surgery these patients have been more likely been planned for a regional anesthesia procedure. Likewise, the advanced age might have tempted the anesthesiologist to classify these patients rather into the RAG group. This assumption can also be assumed if the ASA values of the patients are considered. In the regional anesthesia group the mean ASA value was significantly higher. In considering the risk of anesthesia and the constitution of the patient in terms of ASA quantification, those who were presumably more seriously ill were more likely to receive regional anesthesia. However, we performed a subgroup analysis in an age matched cohort. Even in this subgroup we could not demonstrate a significant difference between the two subgroups either in the cortisol evaluation or in the cognitive assessment.

Our cognitive assessment examined functions that also included executive functions which belong to higher cognitive processes [19]. With increasing age, a decline in the efficiency of executive functions in healthy older people compared to younger people has been reported, along with acute stress aggravates age-related cognitive deficits [20–22]. Some authors hypothesized that higher memory functions with complex cognitive patterns, such as executive functions, are supplied with less energy in an acute stress situation [23]. Furthermore, other studies were able to demonstrate that acute stress partly worsens executive functions [24, 25]. These accompanying impairments can be attributed to a stress-related increased release of catecholamines and glucocorticoids [26]. However, based on our data, although patients in the GA group had higher postoperative cortisol levels, this was not associated with increased POCD.

We assumed that patients scheduled for surgery experience stress in particular. Especially those who are also awake while undergoing surgical procedure and are given local anesthesia, experience additional stressor of an unfamiliar environment. Kazmierski and colleagues were able to demonstrate that after coronary artery bypass graft surgery, increased cortisol levels can lead to cognitive dysfunction [27]. In a non-cardiac-surgical study it was shown that regional anesthesia leads to better neurocognitive test scores than general anesthesia in patients undergoing total knee arthroplasty [28]. In this study the cognitive test values in the regional anesthesia group were unchanged and in the general anesthesia group on the seventh postoperative day cognitive test values were significantly reduced [28]. In our data, the cognitive values ​​in the group undergoing regional anesthesia are also unchanged. However, these findings also apply to the general anesthesia group. This could be explained by the different time of assessment when the cognitive testing was carried out. Our study scheduled the cognitive assessment on the first postoperative day, whereas in the comparative study, the assessment was scheduled on the seventh day.

Although some studies suggested that procedures under regional anesthesia are more suitable in comparison to general anesthesia to avoid POCD, this could not be proven [8]. Thus, available literature has conflicting results regarding anesthesia approaches and the risk of POCD. This could be due to the different surgeries, duration of surgery and anesthesia, a varying patient population and ASA value or different, individual stressors. Rasmussen and co-workers could previously not show any significant differences between regional and general anesthesia with regard to POCD in concordance with our observation [29]. However, Mandal and co-authors showed a significantly reduced cognitive function after general anesthesia compared to a regional anesthesia procedure [30]. A more recent comparative study showed a reduced cognitive function in all study patients, though the magnitude and duration of the cognitive impairment were less pronounced in the patients with epidural anesthesia than in the general anesthesia group [31].

Yuan and co-workers associated an increased risk of cognitive dysfunction to older patients who had elevated salivary cortisol levels preoperatively. They assumed an association between a preoperative neuroendocrine disorder and POCD [32]. The authors also believed that short-term, stress-induced cortisol release could be adapted, whereas chronic stress provokes higher levels of glucocorticoids and could even cause structural brain changes, which leads to cognitive impairment in neurodegenerative diseases [33, 34]. By this background, Mu and colleagues also reported increased cortisol levels on the first postoperative morning to be associated with an increased risk of early postoperative POCD [35]. In addition, it is assumed that an increased postoperative cortisol level leads to a higher risk for the development of delirium [36]. However, this correlation of increased cortisol levels and POCD as well as delirium could not be proven by Glumac and colleagues in a cohort study of cardiosurgical patients according to a strict POCD definition [37]. Likewise, there were no significant differences in the cortisol values ​​in the patients at the sixth postoperative day between the POCD and non-POCD patients after cardiac surgery and general anesthesia. In contrast, in a group of patients of the same age with hip fracture surgery and spinal anesthesia, the baseline plasma cortisol level was the same. However, the POCD patients plasma cortisol levels were significantly increased postoperatively [38]. Moreover, the authors reported that older age and a lower educational level might also be suspected for the occurrence of POCD. In the present study, we also found consistently lower cortisol levels in the regional anesthesia group compared to the general anesthesia group. However, the comparatively higher cortisol levels in the general anesthesia group were not associated with a postoperative cognitive deficit in our study. Even if the subgroup analysis is considered with age matched pairs, the cortisol levels in the GAG were above those in the RAG, but were no longer significantly different and did not show any postoperative cognitive disorders. Furthermore, the only brief increase in cortisol levels observed in the entire general anesthesia group did not lead to any changes in cognitive function. This can possibly be explained by compensatory mechanisms of patients themselves [33]. Additionally, increased cortisol values after stress have been expected in elderly patients [39]. It is conceivable that this elevated cortisol level, which only occurs on the day of the surgery in the general anesthesia group, is accompanied by a stress reaction resulting from fear of the anesthesia. It was shown that the fear of general anesthesia is significantly greater than that of regional anesthesia [40]. However, it was already documented years ago that fear of anesthesia decreases with increasing age [41]. Therefore, this might explain our results of low cortisol levels in regional anesthesia without a peak.

In the current study, we aimed to investigate the influence of regional or general anesthesia and the accompanying stress for the patient with regard to the development of POCD. We used an assessment battery for cognitive evaluation and at the same time determined salivary cortisol on three perioperative days. It was found that the cortisol levels in the regional anesthesia group were lower than in the general anesthesia group. The demographic influencing variables and patients risk factors had no influence on the cortisol level. Although the cortisol levels in the general anesthesia group were significantly higher on the day of surgery in comparison to the regional anesthesia group, this had no influence on the cognitive outcome results. In the cognitive assessment, no differences were found between both groups regarding the difference between pre- and postoperative outcome.

### Limitations

There are some considerable study limitations to the interpretation of these findings. First, average age in our study was 62 years in the GAG and 75 years in the RAG, respectively, which may be still too young to elicit a significant age-related changes in executive function respectively cognitive dysfunction [21]. Secondly, missing cortisol values could also have an alternating effect in the statistics. Missing study data in our data set can be further attributed to insufficient moisture penetration of the cortisol cotton wool. These individually missing cortisol measurements were completely randomly distributed. Thus, no systematic bias can be assumed. Future studies should include larger study population in order to identify small effects of altered cognitive performance. On the other hand, another limitation could arise from the cognitive tests themselves. The cognitive assessment used in the study follows a conservative neuropsychological examination, which is subject to a certain laboratory-like quality such as reliability and validity. However, these assessments cannot be carried out otherwise under standardized conditions. The conditions are therefore also the same for each test person. Nevertheless, it would therefore be conceivable that the influence of stress on cognitive performance could not be detected under standardized assessment conditions. In the light of these considerations, the effects of stress on cognitive performance in everyday life have not been investigated and thus escapes study evaluation. Furthermore, from a cognitive point of view, healthy subjects could not have been challenged sufficiently with the cognitive assessment. Even though the length of the individual tests should not be underestimated, the order of the tests in the second repetition part should possibly have been rearranged in order to prevent a possible habituation effect. Nevertheless, we have used two different individual test versions and limited the test duration in favor of a preserving concentration and in order to avoid a fatigue bias. Schwabe and Wolf demonstrated that an acute stress reaction can affect certain cognitive domains for up to 90 min (e.g. memory retrieval impairment) [42]. However, to what extent this effect can still be demonstrated on the first postoperative day will have to be demonstrated by future studies. This also includes detailed studies on general timing effects and their influence on a surgical stress reaction or the neurobiological effect of pure cortisol secretion on cognitive performance.

## Conclusions

In conclusion, we could not detect any differences in postoperative cognitive outcome between patients receiving regional versus general anesthesia. However, we found a lower cortisol level in the regional anesthesia group. Based on these findings, future studies might further elucidate the impact of different anesthesia regimens on perioperative stress, and its impact on patient outcome.

## Data Availability

The datasets generated and analyzed during the current study are available from the corresponding author on reasonable request.

## Abbreviations

ACE: Angiotensin-convertingenzyme
ADL: activities of daily living
ASA: American Society of Anesthesiologists
FWIT: Color-Word-Interference-Test
GAG: general anesthesia group
POCD: postoperative cognitive disorder
PONV: postoperative nausea and vomiting
QOL: quality of life
RAG: regional anesthesia group
RBMT: Rivermead Behavioral Memory Test
SKT: Short Cognitive Performance Test
TCI: Target Controlled Infusion
